# Sensitive Method for the Quantitative Determination of Risperidone in Tablet Dosage Form by High-Performance Liquid Chromatography Using Chlordiazepoxide as Internal Standard

**Published:** 2013-06

**Authors:** Safwan Ashour, Nuha Kattan

**Affiliations:** Analytical Biochemistry Laboratory, Department of Chemistry, Faculty of Science, University of Aleppo, Aleppo, Syria

**Keywords:** Risperidone, RP-HPLC, bulk powder, pharmaceutical formulations

## Abstract

A selective, sensitive and simple reversed-phase HPLC method for the determination of risperidone in bulk powder and pharmaceutical formulations was developed and validated. Risperidone can be separated on a Supelcosil LC_8_ DB column (250 mm × 4.6 mm i.d., 5 μm particle size) at 40°C with a mobile phase of methanol and 0.1 M ammonium acetate pH 5.50 (60:40, *v/v*), pumped at flow rate 1.0 mL min^-1^ and detected at 274 nm. Chlordiazepoxide hydrochloride was used as internal standard. The retention time of risperidone and chlordiazepoxide hydrochloride was found to be 5.89 and 7.65 min, respectively. The validation of the proposed method was carried out for specificity, linearity, accuracy, precision, limit of detection, limit of quantitation and robustness. The linear range was 4.0-275.0 µg mL^-1^ (r^2^=0.9998) with limits of detection and quantification values of 0.48 and 1.59 μg mL^-1^, respectively. The precision of the method was demonstrated using intra- and inter-day assay RSD values which were less than 3.27%, while the recovery was 99.00-101.12% (*n*=5). According to the validation results, the proposed method was found to be specific, accurate, precise and could be applied to the quantitative analysis of risperidone in raw material and tablets.

## INTRODUCTION

Risperidone, 3-[2-[4-(6-fluoro-1,2-benzisoxazol-3-yl)piperidin-1-yl]ethyl]-2-methyl-6,7,8,9-tetrahydro-4*H*-pyrido[1,2-*a*]pyrimidin-4-one (1), is a benzisoxazole antipsychotic, reported to be an antagonist to dopamine D2 and serotonin (5HT2), adrenergic, and histamine (H1) receptors (2). Few chromatographic methods have been reported in the literature for the analysis of risperidone in pharmaceutical preparations either alone (3, 4), with its degradation products (5) or with other compounds (6, 7). Other techniques for the determination of risperidone from pharmaceutical dosage form have been developed. These techniques include extractive colorimetry (8), chemiluminescence (9), capillary zone electrophoresis (10) and non-aqueous titration (11). There are numerous methods to quantify risperidone and 9-OH-risperidone enantiomers in biological fluids, including HPLC-DAD (12), HPLC with electrochemical detection (13), MEPS–LC–UV (14), LC–MS/MS (15, 16) and affinity capillary electrophoresis and H1 NMR spectroscopy (17). These methods are complicated, costly and time consuming in comparison to a simple HPLC-UV method.

The objective of this work was to develop and validate a sensitive and reliable analytical method using reversed phase-high performance liquid chromatography (RP-HPLC) with low cost of mobile phase, which was used for the first time in this work, for determination of risperidone in raw material and tablets. The method serves as an alternative to the methods described in pharmacopoeias.

## EXPERIMENTAL

### Materials

**Figure 1:**
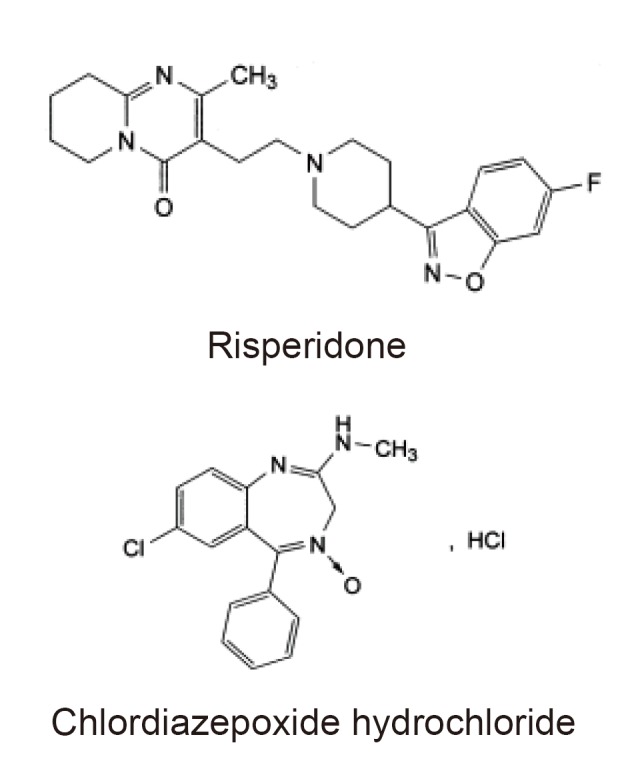
The chemical structure of risperidone and chlordiazepoxide hydrochloride (I.S.).

Working reference standard of risperidone (RSP) and chlordiazepoxide hydrochloride (CDZ) were supplied by Chempi Fine Chemicals (India) and Centaur Pharmaceuticals PVT. Ltd. (India), respectively. The structures of these compounds are shown in Figure 1. HPLC grade methanol and water were purchased from Labscan (Ireland). Analytical reagent grade ammonium acetate (Merck) was used to prepare the mobile phase. Tablets were purchased from Syrian market, containing risperidone 1, 2 and 4 mg per tablet.

### HPLC system and Chromatographic conditions

The chromatographic system consisted of Hitachi (Japan) Model L-2000 equipped with a binary pump (model L-2130, flow rate range of 0.000-9.999 mL min^-1^), degasser and a column oven (model L-2350, temperature range of 1-85°C). All samples were injected (10 μL) using a Hitachi L-2200 auto sampler (injection volume range of 0.1-100 μL). Elution of all analytes were monitored at 274 nm by using a Hitachi L-2455 absorbance detector (190-900 nm) containing a quartz flow cell (10 mm path and 13 μL volume). Separation was achieved on Supelcosil LC_8_ DB column (250 mm × 4.6 mm i.d., 5 μm particle size). The mobile phase was a mixture of an ammonium acetate solution (0.1 M, pH 5.50) and methanol (40:60, *v/v*) and was filtered and degassed by ultrasonic agitation before use. The mobile phase was prepared weekly and was delivered at a flow rate of 1.0 mL min^-1^. Data were monitored and processed using automation system software. Peak areas were integrated automatically by computer using the Ezchrom Elite Hitachi software program. The system was operated at 40°C.

### Standard solutions

Standard stock solution of RSP (1.0 mg mL^-1^) was prepared by direct weighing of standard substance with subsequent dissolution in methanol. Stock standard solution of chlordiazepoxide (0.5 mg mL^-1^) was prepared by dissolving appropriate amount of the compound in methanol. A series working standard solutions of RSP (4.0-275.0 µg mL^-1^) were prepared by diluting the stock standard solution with the methanol. In each sample 0.5 mL of CDZ was added (25 µg mL^-1^ in the final volume 10 mL). These solutions were stored in the dark at 2-8°C and found to be stable for one month at least.

### Calibration graphs

To construct the calibration curve five replicates (10 μL) of each standard solution were injected immediately after preparation into the column and the peak area of the chromatograms were measured. Then, the mean peak area ratio of RSP to that of the internal standard was plotted against the corresponding concentration of RSP (4.0-275.0 µg mL^-1^) to obtain the calibration graph.

### Assay for dosage forms

Twenty tablets containing RSP were weighed and finely powdered. Portions of the powder (each equivalent to the weight of five tablets) were accurately weighed into 50 mL volumetric flasks and 30 mL methanol was added. The volumetric flasks were sonicated for 15 min to effect complete dissolution of RSP, the solutions were then made up to volume with methanol. The sample solutions were filtered through 0.45 μm nylon filter. The aliquot portions of the filtrate were further diluted to get final concentration of 100 µg mL^-1^ in the presence of 25 µg mL^-1^ of internal standard. Finally, 10 μL of each diluted sample was injected into the column and chromatogram was recorded. Peak area ratios of RSP to that of CDZ were then measured for the determination. RSP concentrations in the samples were then calculated using peak data and standard curves.

## RESULTS AND DISCUSSION

### Optimization of the chromatographic conditions

**Figure 2:**
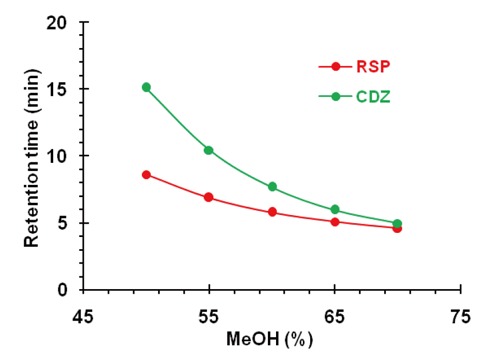
Plots of the retention time vs. methanol percentage in the mobile phase of RSP and CDZ.

**Figure 3:**
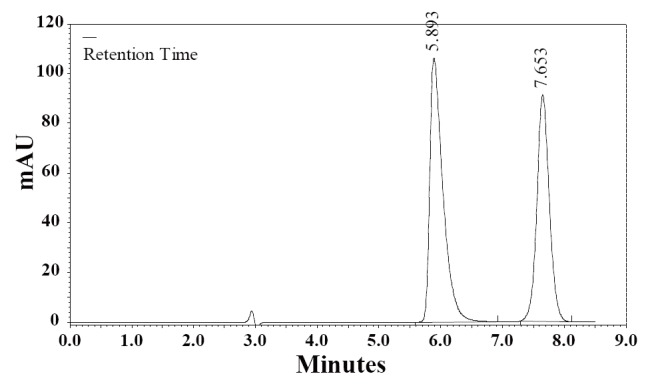
A typical chromatogram of a mixture of RSP (100 μg mL^-1^) and the internal standard, CDZ, (25 μg mL^-1^) in the mobile phase. Chromatographic conditions: RP-HPLC on Supelcosil LC_8_ DB; mobile phase: NH_4_OAc (0.1 M, pH 5.5) and methanol (40:60, v/v); flow rate 1.0 mL min^-1^ and detection at 274 nm.

Separation of ionizable analytes-acids and bases in terms of column efficiency, selectivity and retention depends on the pH of the mobile phase. Retention is usually improved on a non-polar column, by changing pH so that analytes are separated in their un-ionizable forms. Also, interaction between analyte and the silica surface of the column packing, that causes poor peak shape, could be minimized by choosing appropriate composition and pH of the mobile phase. To choose the optimal pH for risperidone separation it is necessary to take into account that the nitrogenous drugs are present in positively charged protonated forms, thus risperidone may exist in solution in cationic, neutral, zwitterionic and anionic forms. Relative percentages of these forms in the solution depend upon the pH of the solution. The effect of pH in the chromatographic elution of the compounds was investigated by change the concentration values of the aqueous component of the mobile phase from 4.0 to 6.5. As pH was reduced below 4.0 risperidone was eluting earlier and at pH 6.5 it is eluting at retention time of 7.11 min and merging with chlordiazepoxide peak. Fifty degree celsius was a compromise, because at 50°C the peaks were narrower, but column life was rather short. At 40°C column temperature and pH 5.50 of aqueous component of the mobile phase, the peak shape of RSP and CDZ was found symmetrical. To optimize the HPLC parameters, different columns (Supelcosil LC_8_ DB 250 mm × 4.6 mm i.d., 5 µm particle size); (ODS Hypersil C_18_ 250 mm × 4.6 mm i.d., 5 μm particle size) and several mobile phase compositions were tried. Supelcosil LC_8_ DB column (250 mm × 4.6 mm i.d., 5 µm particle size) gave the minimal elution time with good resolution. The effect of composition of the mobile phase on the retention time of RSP and the internal standard, CDZ, was investigated. Results of the effect of methanol percentage in the mobile phase are presented in Figure 2. An increase in the percentage of methanol decreases the retention of RSP. Increasing methanol percentage to more than 70% RSP peak is eluted with the solvent front, while at methanol percentage lower than 50% the elution of CDZ peak is seriously delayed. A satisfactory separation and peak asymmetry for the drug was obtained with mobile phase consisting of methanol: 0.1 M NH_4_OAc pH 5.50 (60:40, *v/v*), pumped at a flow rate 1.0 mL min^-1^ at 40°C. Quantitation was achieved with UV detection at 274 nm based on peak area. A representative chromatogram is shown in Figure 3. The retention time of RSP and CDZ were 5.89 and 7.65 min, respectively.

### Validation of the method

**Figure 4:**
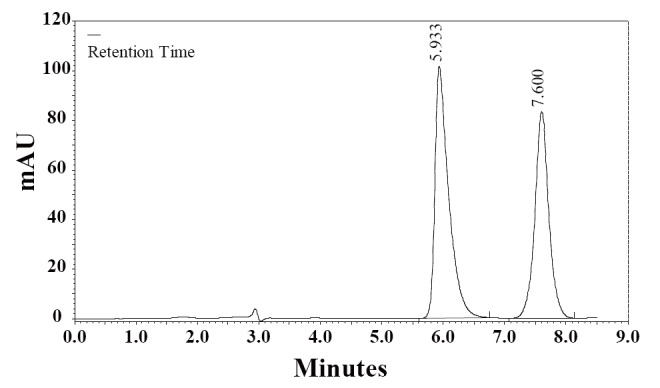
A chromatogram of RSP (100 μg mL^-1^) prepared from risperdine tablet in the mobile phase in the presence of the internal standard, CDZ, (25 μg mL^-1^). Chromatographic conditions: RP-HPLC on Supelcosil LC_8_ DB; mobile phase: NH_4_OAc (0.1 M, pH 5.5) and methanol (40:60, v/v); flow rate 1.0 mL min^-1^ and detection at 274 nm.

**Specificity.** The specificity of the HPLC method is illustrated in Figure 3 where complete separation of RSP and CDZ was noticed. The HPLC chromatogram recorded for the analyte in tablet (Fig. 4) showed almost no peaks within a retention time range of 20 min. The figures show that RSP is clearly separated and the peak of analyte was pure and excipients in the formulation did not interfere the analyte. Thus, the HPLC method presented in this study is selective for RSP.

**Linearity and limits of quantitation and detection.** The calibration curve of RSP was linear (r^2^>0.999) over the concentration range 4.0–275.0 µg mL^-1^. Straight line for RSP was obtained, when the area of the peaks was plotted versus concentration (Table 1). Also, Linear relationship was obtained between the peak area ratio of RSP to that of the internal standard CDZ and the corresponding concentration of RSP (4.0–275.0 µg mL^-1^), as shown by the equations presented in Table 1. The minimum level at which the investigated compounds can be reliably detected (limit of detection, LOD) and quantified (limit of quantitation, LOQ) were determined experimentally. LOD were expressed as the concentration of drug that generated a response to three times of the signal to-noise (S/N) ratio, and LOQ was 10 times of the S/N ratio. The LOD of RSP attained as defined by IUPAC (18), LOD_(*k*=3)_ = *k*× *S*_a_/*b* (where *b *is the slope of the calibration curve and *S*_a_ is the standard deviation of the intercept), was found to be 0.48 μg mL^-1^. The LOQ were also attained according to the IUPAC definition, LOQ_(*k*=10)_ = *k*× *S*_a_/*b*, and was found to be 1.59 μg mL^-1^.

**Table 1:** Calibration data for the estimation of risperidone by HPLC

Parameter	Risperidone

Optimum concentration range (μg/mL)	4.0-275.0
Regression equation^a^	*A*_RSP_ = 0.6113*C*_RSP_ + 0.9291
Correlation coefficient (r^2^)	0.9998
Standard deviation of slope	0.0008
Standard deviation of intercept	0.07
Regression equation^b^	*R*_RSP/CDZ_= 0.0112*C*_RSP_ + 0.0171
Correlation coefficient (r^2^)	0.9999
Standard deviation of slope	5.4×10^-5^
Standard deviation of intercept	0.002
Limit of quantification, LOQ (μg/mL)	1.59
Limit of detection, LOD (μg/mL)	0.48

a Regression equation for the peak area of RSP *vs*. concentration of RSP in μg/mL;

b Regression equation for the ratio of peak area of RSP to that of CDZ *vs*. concentration of RSP in μg/mL.

**System suitability.** The system suitability was determined by making five replicate injections and analyzing each solute for their peak area, resolution and peak tailing factor. The system suitability requirements for 100.0 µg mL^-1^ of RSP in the presence of 25.0 µg mL^-1^ of internal standard was a %RSD for peak area less than 0.51, a peak tailing factor less than 1.5 and an *R*s greater than 4.0 between adjacent peaks. This method met these requirements. The results are shown in Table 2.

**Table 2:** System suitability parameters

Parameters	Risperidone	Chlordiazepoxide hydrochloride

Theoretical plates (*N*)	3790	6927
Resolution factor (*Rs*)	-	4.69
Tailing factor (*T*)	1.30	1.05
Capacity factor (*k*)	4.88	6.64
% RSD for five injections	0.17	0.51

**Accuracy and precision.** The precision and accuracy of the method were evaluated by intra- (analysis of standard solutions of RSP in replicates of five in the same day) and inter-day (analysis of standard solutions of RSP in replicates of five on 3 different days from day 1 to 30 after preparation) assay variance (Table 3). The standard deviation, relative standard deviation, recovery and relative percentage error of different amounts tested were determined, as recorded in Table 3. The accuracy of the method is indicated by the excellent recovery (99.00-101.12%) and the precision is supported by the low standard deviation. Table 3 shows that the percent error of the method was always less than 1.12%; therefore, it was concluded that the procedure gives acceptable accuracy and precision for the analyte.

**Table 3:** Accuracy and precision of within and between run analysis for the determination of risperidone by HPLC

Nominal concentration (μg/mL)	Risperidone			
	Mean ± SD (μg/mL)	RSD (%)	Recovery (%)	Relative error (%)

*Intra-day (n=5)*				
4.00	3.96 ± 0.08	2.02	99.00	-1.00
10.00	10.07 ± 0.19	1.88	100.70	0.70
25.00	25.20 ± 0.26	1.03	100.80	0.80
50.00	50.15 ± 0.37	0.74	100.30	0.30
100.00	100.04 ± 0.60	0.60	100.04	0.04
200.00	201.38 ± 0.52	0.26	100.69	0.69
275.00	275.17 ± 0.62	0.22	100.06	0.06
*Inter-day (n=5)*				
4.00	3.97 ± 0.13	3.27	99.25	-0.68
10.00	10.05 ± 0.18	1.79	100.50	0.50
25.00	25.09 ± 0.39	1.55	100.36	0.36
50.00	50.56 ± 0.65	1.28	101.12	1.12
100.00	100.37 ± 0.93	0.93	100.37	0.37
200.00	201.21 ± 1.39	0.69	100.60	0.60
275.00	276.21 ± 0.82	0.29	100.44	0.44

**Robustness.** The robustness of an analytical procedure is a measure of its capacity to remain unaffected by small, but deliberate variations in method parameters and provides an indication of its reliability during normal usage. Robustness of the method was investigated under a variety of conditions including changes of pH of the mobile phase, flow rate, percentage of methanol in the mobile phase and column oven temperature. The standard solution is injected in five replicates and sample solution of 100% concentration is prepared and injected in triplicate for every condition and % R.S.D. of assay was calculated for each condition. The degree of reproducibility of the results obtained as a result of small deliberate variations in the method parameters has proven that the method is robust (Table 4).

**Table 4:** Results of robustness study

Factor	Level	Risperidone	
		Mean % assay (*n*=5)	% RSD of results

pH of mobile phase	5.4	100.3	1.32
	5.6	100.1	0.86
Flow rate (mL/min)	0.9	99.9	0.97
	1.1	100.2	0.58
Column oven temperature (°C)	35	99.7	1.24
	45	100.6	0.47
% of methanol	55	100.4	0.86
	65	100.8	0.53

**Stability studies.** Stability studies were carried out at laboratory temperature for a month to find potential stability problems of the drug in the formulations. Samples were analyzed at intervals of 0, 1, 5, 15 and 30 days. The results obtained are given in Table 5. The percent RSD values between subsequent readings gave an indication of the stability of the drug in the formulations.

**Table 5:** Stability study for the drug in different formulations

Formulations	Time (days)	Amount found^a^ (mg)	% Recovery	% ± RSD

Risperdine (4 mg/tablet)	0	4.05	101.27	0.36
	1	4.06	101.50	0.44
	5	4.04	101.00	0.39
	15	4.07	101.75	0.51
	30	4.02	100.50	0.32
Zophrenal (4 mg/tablet)	0	4.06	101.50	0.98
	1	4.09	102.25	0.86
	5	4.02	100.50	0.48
	15	4.05	101.27	0.64
	30	4.04	101.00	0.52
Risperdine (2 mg/tablet)	0	2.01	100.50	0.49
	1	2.00	100.00	0.39
	5	1.99	99.50	0.72
	15	2.02	101.00	0.62
	30	2.03	101.50	0.50
Risperid fort (2 mg/tablet)	0	2.05	102.50	0.43
	1	2.03	101.50	0.72
	5	2.04	102.00	0.48
	15	2.02	101.00	0.36
	30	2.01	100.50	0.32
Risperid (1 mg/tablet)	0	1.02	102.00	0.26
	1	1.01	101.00	0.40
	5	0.98	98.00	0.45
	15	0.99	99.00	0.37
	30	1.01	101.00	0.26

a Five independent analyses.

### Application of the assay

The developed method was applied for the determination of RSP in tablets from the Syrian market. Figure 4 shows an HPLC chromatogram of RSP in tablets. The results obtained with the proposed method were compared with the official method (11). The results in Table 6 indicate the high accuracy and precision. As can be seen from Table 6, the proposed method has the advantages of being virtually free from interferences by excipients such as glucose, lactose and starch or from common degradation products. The results obtained were compared statistically by the Student’s *t*-test (for accuracy) and the variance ratio *F*-test (for precision) with those obtained by the official method for the samples of the same batch (Table 6). The values of *t*- and *F*-tests obtained at 95% confidence level did not exceed the theoretical tabulated value indicating no significant difference between the methods compared.

**Table 6:** Determination of RSP in tablets formulations by the proposed and official methods

Sample	Recovery (%)^a^ ± S.D.	
	Risperidone	
	Proposed method	Official method

Pure	100.08 ± 0.23	99.52 ± 0.29
*t*-value	1.49	
*F*-value	1.59	
Risperdine (4 mg/tablet)- Medico Labs (Syria)		
Mean ± S.D.^a^	101.27 ± 0.37	102.60 ± 0.47
*t*-value^b^	1.49	1.63
*F*-value^b^	1.61	
Zophrenal (4 mg/tablet)- Ubari Phrma (Syria)		
Mean ± S.D.^a^	101.60 ± 0.13	100.92 ± 0.18
*t*-value^b^	1.83	1.95
*F*-value^b^	1.92	
Risperdine (2 mg/tablet)- Medico Labs (Syria)		
Mean ± S.D.^a^	100.52 ± 0.50	100.49 ± 0.61
*t*-value^b^	2.29	2.17
*F*-value^b^	1.49	
Risperid fort (2 mg/tablet)- Racha Labs (Syria)		
Mean ± S.D.^a^	102.43 ± 0.44	101.06 ± 0.53
*t*-value^b^	2.03	1.91
*F*-value^b^	1.45	
Risperid (1 mg/tablet)- Racha Labs (Syria)		
Mean ± S.D.^a^	102.79 ± 0.26	101.63 ± 0.31
*t*-value^b^	1.79	1.69
*F*-value^b^	1.42	

a Five independent analyses;

b Theoretical values for *t* and *F*-values at five degree of freedom and 95% confidence limit are *t*=2.776 and *F*=6.26.

## CONCLUSION

A specific, precise and sensitive RP-HPLC method has been developed and validated for quantitative determination of resperidone in tablet formulation with limits of quantitation of 1.59 μg mL^-1^ and detection of 0.48 μg mL^-1^. The sample recoveries from all formulations were in good agreement with their respective label claims, which suggested non-interference of formulation excipients in the estimation. The developed method is more speed and higher sensitivity as compared to sophisticated spectrophotometric techniques and similar reported methods and has a wider range of linearity. Moreover, the lower solvent consumption along with the short analytical run time of 8.0 min leads to an environmentally friendly chromatographic procedure, which makes it especially suitable for routine quality control analysis work.
